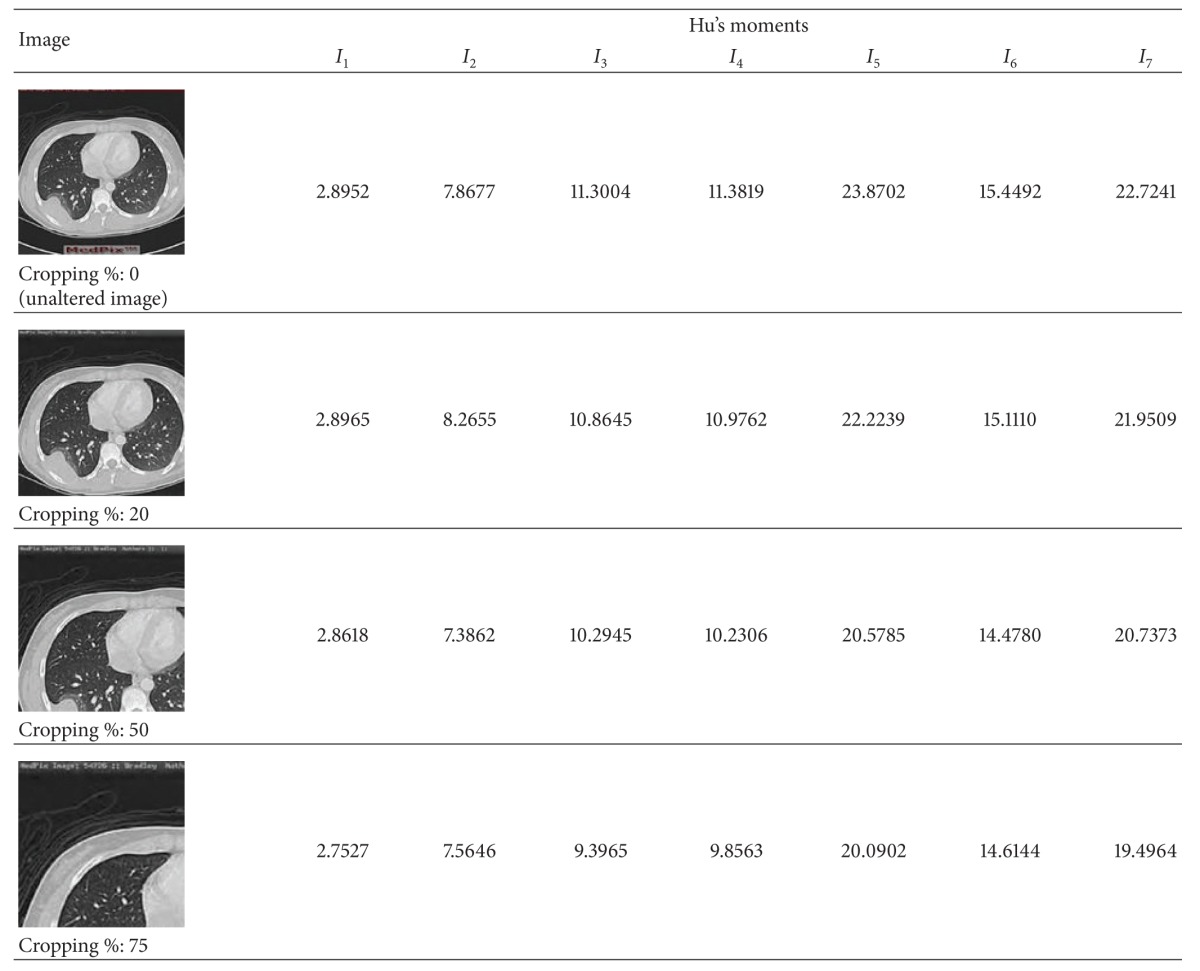# A QR Code Based Zero-Watermarking Scheme for Authentication of Medical Images in Teleradiology Cloud

**DOI:** 10.1155/2013/516465

**Published:** 2013-07-18

**Authors:** V. Seenivasagam, R. Velumani

**Affiliations:** ^1^Department of Computer Science and Engineering, National Engineering College, Kovilpatti 628503, India; ^2^Department of Information Technology, Sethu Institute of Technology, Pulloor, Kariapatti 626115, India

## Abstract

Healthcare institutions adapt cloud based archiving of medical images and patient records to share them efficiently. Controlled access to these records and authentication of images must be enforced to mitigate fraudulent activities and medical errors. This paper presents a zero-watermarking scheme implemented in the composite Contourlet Transform (CT)—Singular Value Decomposition (SVD) domain for unambiguous authentication of medical images. Further, a framework is proposed for accessing patient records based on the watermarking scheme. The patient identification details and a link to patient data encoded into a Quick Response (QR) code serves as the watermark. In the proposed scheme, the medical image is not subjected to degradations due to watermarking. Patient authentication and authorized access to patient data are realized on combining a Secret Share with the Master Share constructed from invariant features of the medical image. The Hu's invariant image moments are exploited in creating the Master Share. The proposed system is evaluated with Checkmark software and is found to be robust to both geometric and non geometric attacks.

## 1. Introduction

Teleradiology enables medical images to be transmitted over electronic networks for improved clinical interpretation, healthcare access, archiving, and research. Recently, teleradiology services are utilized by healthcare institutions for real-time emergency radiology services, in the absence of onsite radiologists. In a case study by Liu and Zhang [1] on security of teleradiology systems, the security requirements for providing teleradiology services to multiple healthcare organizations are identified. This paper emphasizes the importance of deploying a mechanism for a positive detectable binding between patient identification information and medical records. The need for patient authentication in remote health monitoring is emphasized by Sriram et al. [2]. The authors propose an EGC and accelerometer based system to uniquely identify the patients for administering remote healthcare. Medical image watermarking has been proposed as a promising solution for authentication in many parts of the literature. The application of watermarking techniques for authentication and protection of medical images is discussed in a paper by Coatrieux et al. [3]. A reversible [4] watermarking scheme for authentication of Digital Imaging and COmmunications in Medicine (DICOM) images is proposed by Al-Qershi and Khoo. In this scheme, patient data is embedded in the Region of Interest (ROI) and data required for tamper detection and recovery is embedded in the Region of NonInterest (RONI). Watermarking techniques in spatial and transform domains such as Discrete Cosine Transform (DCT), Discrete Wavelet Transform (DWT) are thoroughly investigated in a survey article by Rey and Dugelay [5]. The Contourlet Transform (CT) domain has attracted the attention of researchers with its directionality and anisotropy properties in addition to multiscale and time-frequency localization proprieties of wavelets. This transform provides the best approximation of smooth contours and edges of the image subjected to decomposition. Many authors have implemented blind and nonblind watermarking algorithms in the contourlet domain [6–8]. Many transform based watermarking algorithms have been proposed in combination with Singular Value Decomposition (SVD). The Singular Values (SVs) are suitable for watermarking due to their stability and representation of intrinsic algebraic properties of images. Watermarking schemes in composite domains such as DCT-SVD [9] and DWT-SVD [10] perform SVD in the candidate subbands for watermarking. In a nonblind CT-SVD [11] algorithm, CT and SVD transforms are applied on the Low Frequency (LF) subbands of both the host image and watermark. SVs of the host image are modified by the SVs of the watermark image.

The conventional watermarking systems which embed the watermark in the spatial, frequency, or hybrid domains suffer from the tradeoff between the conflicting requirements of capacity, transparency, and robustness. Zero-watermarking [12] or nonwatermarking has emerged as a new paradigm of watermarking which eliminates the imperceptibility issues due to watermark embedding. This approach does not embed a watermark into the host image physically, whereas it is logically embedded. The watermark embedding is analogous to creation of a Master Share and Secret Share out of the host image and a watermark image at the sender's end. Similarly, extraction refers to the reconstruction of the watermark by combining the Master Share and Secret Share at the receiver's end. The zero-watermarking approach exploits the essential invariant characteristics of the host image to construct the Master Share at both the ends. A zero-watermarking scheme for medical images in the DCT domain proposed by Dong et al. [13] combines visual feature vectors, encryption, and third party authentication to address security, confidentiality, and integrity issues. Similarly, another zero-watermarking scheme for medical images, in which the sign sequence of the Discrete Fourier Transform (DFT) coefficients of the host image is taken as the feature vector to achieve robustness, is also presented by Dong et al. [14].

Over the past decade, 2D QR codes have gained popularity in the authentication of different commodities including multimedia data. The QR code was introduced by Denso-Wave [15] in 1994 to keep track of vehicle parts. Ease of generation of QR codes with free software and the penetration of smart phones enabled with QR code readers have made them widely applicable in different fields including manufacturing industries, shipping, airline, healthcare, advertising, and entertainment. The QR codes encoded with patient's data on their wristbands enable the hospitals to identify the patients and administer appropriate clinical procedures. Medication lists, treatment plans, appointment dates, contact details, and referral information of a patient can be encoded into a QR code. A QR code based authentication scheme is proposed by Liao and Lee [16], as an alternate for one-time password authentication scheme, for a remote user to access services from a service provider.

 In this paper, we present a general framework for patient authentication and controlled access to Electronic Health Records (EHR) in a teleradiology environment. It is based on a zero-watermarking scheme for authentication of medical images with a 2D QR code which encodes the patient identification data. We have chosen the hybrid CT-SVD domain for watermarking; the watermark can be constructed by the authorized personnel only on possession of the Secret Share. 

The rest of the paper is organized as follows.  Section 2 covers the background of this work in 4 subsections. The approaches followed in the proposed system are discussed in Section 3. The proposed system is given in Section 4, followed by experimental results and discussions in Sections 5 and 6, respectively. The paper is concluded in Section 7.

## 2. Background

### 2.1. Patient Authentication in Cloud Based Teleradiology

Medical images are generally watermarked to address security issues such as authenticity, integrity, and confidentiality. We understand from the survey article of Navas and Sasikumar [17] that security of medical images can presumably be achieved by embedding additional data into medical images through digital watermarking. According to Li et al. [18], cloud based medical image exchange simplifies image storing, archiving, sharing and accessing services between radiologists, referral hospitals, physicians, and specialists online. Hospitals that deploy cloud based medical image exchange can view and share images and reports with their referral partners in real time, without relying on physical storage media. Medical image sharing through the cloud obviously eliminates duplication of tests and exposure to radiations and ensures patient safety. The need for diverse security and privacy requirements in healthcare institutions on deployment of teleradiology practices is addressed in a paper by Shini et al. [19]. These requirements are governed by legislative regulations such as Health Insurance Portability and Accountability Act (HIPAA). The standards for protection and privacy of individually identifiable health information and disclosure have been defined in HIPAA. According to the standards framed by Cramer et al. [20] for Canadian Association of Radiologists (CAR), the remote radiologist must identify the patient unambiguously with personally identifiable attributes such as patient name, identification number, date and time of examination, institution of origin, nature of examination, and brief patient history. The standard also says that this information should accompany the image file or may also be transmitted by other secure means such as fax or email.

Transfer of radiology information and Personal Health Information (PHI) of the patients to remote reading sites poses severe security risks. Particularly, data authentication and integrity are essential requirements in teleradiology. Embedding patient-specific metadata as watermark into the medical image is a sensible solution towards imparting authentication. The embedded watermark can be extracted to verify the identity of the patient, and the extracted metadata can augment the cover medical image for a thorough diagnosis. A review paper by Nyeem et al. [21] that explores the requirements of watermarking techniques in teleradiology justifies the application of watermarking techniques for attaining the primary objectives of origin authentication and content authentication. With the evolution of the dayhawk and nighthawk radiology services, remote radiologists examining the clinical images may need to access the past medical history of the patient for a thorough study. The paradigm of nighthawk radiology services and the need to push these data through fax, emails, and telephone calls are discussed by Benjamin et al. [22].

 Further, the Health Information Technology for Economic and Clinical Health (HITECH) Act enacted in 2009 includes provisions to protect patient data. Sarrail and Stromberg [23] present the implications of this act on healthcare services and its stipulations to trace breaches involving healthcare organizations, their business associates, and service providers. The authors advocate smart card technology based solutions for authentication, data security, and access control.

### 2.2. QR Code Based Authentication

**Figure 1:**
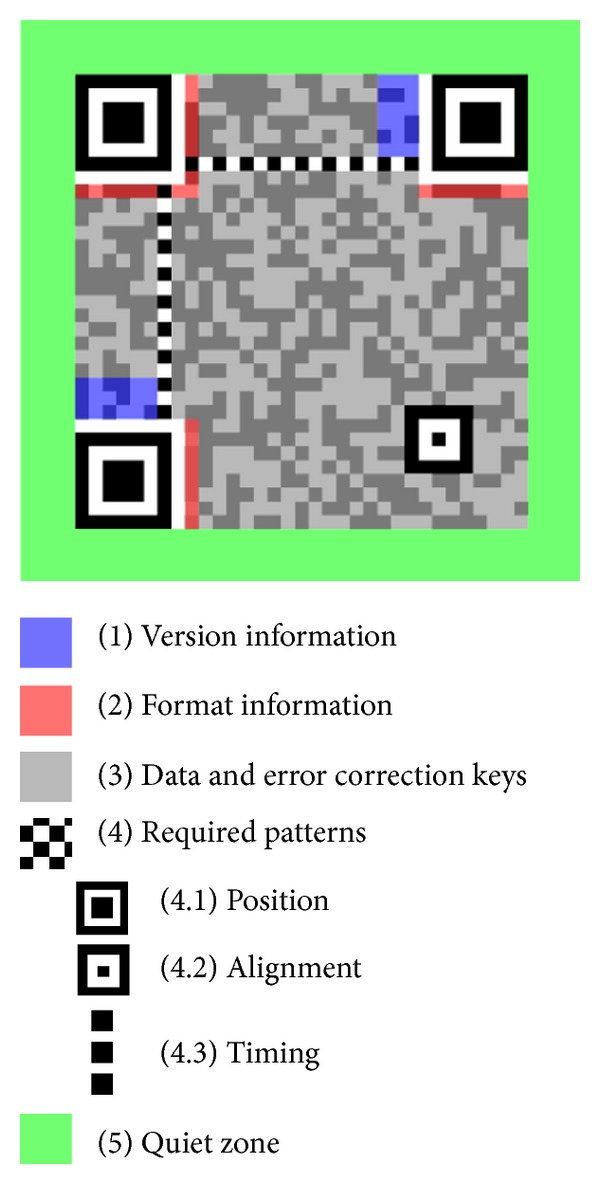
Structure of QR Code. http://en.wikipedia.org/wiki/File:QR_Code_Structure_Example_2.svg.

A QR code exhibits attractive features such as high capacity encoding of data, small printout size, Chinese and Japanese character representation, resistance to dirt and damage, readability from any direction in 360 degrees, and varied error correction levels. The structure of the QR code is shown in Figure 1.

In large healthcare organizations, 2D codes encoded in the wrist bands ensure positive patient identification right from admission to transfer. Many commercial [24] healthcare solution providers offer 2D barcode technologies for different classes of patients.

The use of 1D, 2D, and Radio Frequency IDentification (RFID) based codes in patient identification is elaborately reviewed by García-Betances and Huerta [25]. The authors conclude that QR codes are ideal for patient identification and quick remote access of electronic patient records. The use of QR codes for instant access to patient's medication information by emergency workers is discussed in an article by Davis [26]. The necessary data for emergency care are provided by the patients in the healthcare institution's website, and the links encoded into QR codes are placed as stickers in their wrist bands, for access by paramedics on emergency. The concept of authentication of multimedia [27] content with a QR code is proposed by Kim et al. The authentication mechanism proposed in this paper encodes the Universal Content Identifier (UCI) of the digital content into a 2D barcode and invisibly embeds it into the host Image in the spatial and transform domains. 

### 2.3. Zero-Watermarking Schemes

Direct embedding of watermarks within host images introduces obvious visual degradations and artifacts which are hindrances to analysis of medical and forensic images. The imperceptibility issues are completely eliminated in zero-watermarking schemes. In a scheme proposed by Chang et al. [28], the host image is partitioned into nonoverlapping blocks, and a binary pattern is created out of the variances of the blocks. A secret key is generated out of an XOR operation between the binary pattern and the binary watermark. During extraction, the secret key is XORed with the binary pattern extracted from the host image to recover the watermark. In a vector quantization based watermarking system proposed by Charalampidis [29], a binary pattern is created out of the similarity characteristics of neighboring blocks of natural images. In a scheme proposed by Sang et al. [30], differences in intensity values of the pixels in the host image are compared with the output values of a spatial domain based neural network to generate the binary pattern. 

Zero-watermarking schemes based on Visual Cryptography (VC) for copyright protection are proposed in many papers. In VC based schemes, the watermarks are extracted by the human visual system on stacking the Master and Secret Shares. In the scheme proposed by Hsu and Hou [31], the sampling distribution of means for a normal population is employed to create a Master Share from the host image. The Master Share is created from the composite DWT_SVD domain in a scheme proposed by Wang and Chen [32]. A hybrid scheme proposed by Rawat and Raman in [33] applies Fractional Fourier Transform (FrFT) and SVD on the nonoverlapping blocks of the host image to generate the Master Share. The Secret Share is generated from the Master Share and the secret watermark image on applying the rules of visual cryptography. 

Recently, another zero-watermarking scheme based on visual secret sharing is proposed by Fan et al. [34]. This scheme employs the Bose-Chaudhuri-Hocquenghem (BCH) code for error correction. The Master Share is created from the most significant bit planes of the host image. DWT is applied to the image matrix comprising the selected bit planes, and the coefficients of the Low-Low (LL) subband are randomly selected with a secret key to form the Master Share. The Secret Share is created from the master matrix, quantized host image, and the scrambled watermark. During extraction, Master Share is created from the host image following a similar procedure and is combined with the Secret Share to extract the watermark. 

### 2.4. Contourlet and SVD Transform Domain

**Figure 2:**
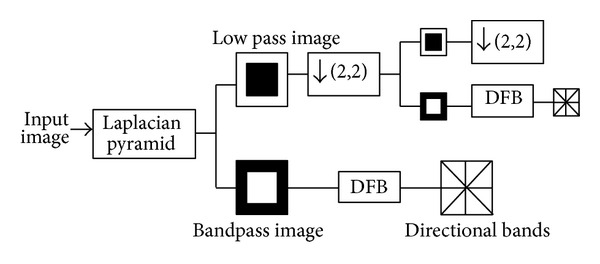
Contourlet Decomposition.

Watermarking algorithms in the composite CT-SVD domain improve the transparency and robustness. The Contourlet Transform (CT) proposed by Do and Vetterli [35] combines both Laplacian Pyramid (LP) and Directional Filter Bank (DFB) structure. The framework for Contourlet decomposition is given in Figure 2. 

Singular Value Decomposition is a linear algebraic tool widely used in factorization and approximation of matrices. For any *n* × *n* real or complex matrix *A*, SVD is a factorization of the form given as follows:

(1) $$\begin{array}{c} \;\left[U,S,V\right]=\text{SVD}\left(A\right), \end{array}$$

where *S* is a *n* × *n* rectangular diagonal matrix with nonnegative real numbers on the diagonal and *U* and *V* are the unitary matrices of the order *n* × *n*. The diagonal entries *S*_*i*,*i*_ of *S* are known as the SVs of *A*. The columns of *U* and *V* are called as left-singular vectors and right-singular vectors of *A*, respectively. Matrix *A* can be reconstructed from the singular and unitary matrices as shown in the following:

(2) $$\begin{array}{c} A=U*S*V^{\prime }, \end{array}$$

where *V*′ is the complex conjugate of *V*.

 The singular values of *S* matrix are invariant to transpose, flipping, scaling, rotation, and translation. Smaller modifications to the images do not significantly change their singular values. Further, best approximation of an image can be realized with only a few significant singular values. The composite CT-SVD domain provides better robustness to different classes of attacks. A zero-watermarking scheme proposed by Zeng and Zhou [36] embeds the watermark in the largest SVs of the nonoverlapping blocks of the LF subband in the Contourlet domain. This scheme is reported to be robust against attacks such as added noise, JPEG compression, and cropping.

## 3. Materials and Methods

In this section we present the methods followed in implementing the system. The subsections cover watermark generation, representation of image features with Hu invariant moments, and Triangular Number Generation function for watermark embedding and extraction.

### 3.1. Watermark Generation

Health Level 7 (HL7) defines clinical standards and message formats and standard frameworks for representation and exchange of clinical information between healthcare institutions. The Patient IDentification (PID) [37] segment is an important component of the HL7 Admission, Discharge & Transfer (ADT) message that contains the unique identification data of the patient. It has 30 different fields including patient ID number, Patient Name, Date/Time of Birth, Race, Patient Address, Sex, Social Security Number, and so forth, which are sufficient to unambiguously identify a patient. The entire list of patient identifiable attributes and a sample PID appears in http://www.corepointhealth.com/resource-center/hl7-resources/hl7-pid-segment.

**Figure 3:**
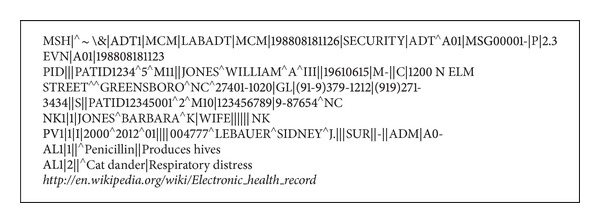
Watermark contents.

In the proposed system, we have taken this sample HL7 Patient IDentification segment (HL7 PID) augmented with the Universal Resource Locator (URL) string of a EHR as the watermark. The watermark contents are shown in Figure 3. The sample URL for EHR is shown in italic. 

**Figure 4:**
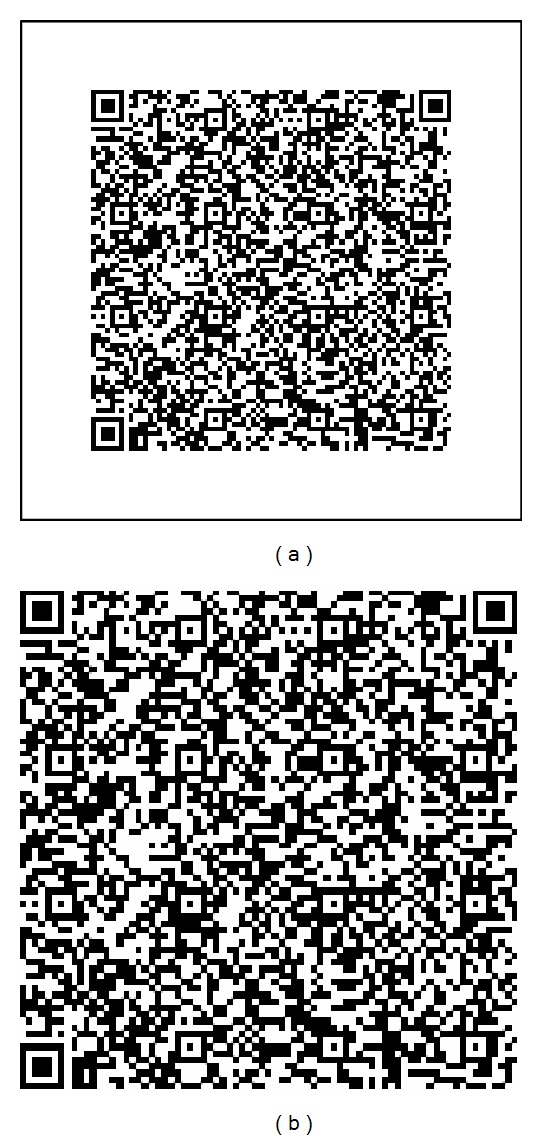
(a) QR code of watermark contents. (b) Trimmed QR code.

The patient identification data is encoded into a QR code with the Zxing [38] QR code generator available at http://zxing.appspot.com/generator. The generated QR code of size 120 × 120 is resized to 128 × 128. Further, to reduce the computational overheads, the watermark is trimmed by eliminating the white region which is called the quiet zone. The size of the resultant watermark is 77 × 77. The original and the trimmed watermarks are shown in Figures 4(a) and 4(b). The bounding rectangle around the quiet zone of Figure 4(a) is not part of the QR code generated; it is drawn to define the boundary of the QR code only.

### 3.2. Hu Invariant Moments

Robustness in zero-watermarking system is attributed to the Master Share that represents the essential features of the host image. It is also elemental in construction of the Secret Share according to the principles of zero-watermarking system. In this system we have employed the Hu's [39] invariant moments to create the master share. Hu introduced a set of 7 orthogonal image moments of which the first 6 are invariant to affine transformations and the 7th is to distinguish mirrored images. Many robust watermarking schemes have been proposed based on image moments. In the schemes proposed by Alghoniemy and Tewfik [40, 41], invariant watermarks are generated out of the image invariant moments and they are reported to be robust to both geometric and nongeometric attacks. Given a 2D image *f*(*x*, *y*), the Hu's invariant orthogonal moments are computed as below.

The 2D moment of order (*p* + *q*) of a digital image*f*(*x*, *y*) of size *M* × *N* is defined as

(3) $$\begin{array}{c} m_{pq}=\sum_{x=0}^{M-1}\sum_{y=0}^{N-1}x^{p}y^{q}f\left(x,y\right), \end{array}$$

where *p* = 0,1, 2,…, *M* − 1 and *q* = 0,1, 2,…, *N* − 1 are integers. The corresponding central moment of order (*p* + *q*) is defined as

(4) μpq=∑x=0M−1∑y=0N−1(x−x¯)p(y−y¯)qf(x,y)for  p=0,1,2,…,M−1,  q=0,1,2,…,N−1,

where

(5) $$\begin{array}{c} \bar{x}=\frac{m_{10}}{m_{00}},\;\;\bar{y}=\frac{m_{01}}{m_{00}}. \end{array}$$

The normalized central moment of order (*p* + *q*) is defined as

(6) $$\begin{array}{c} \eta_{pq}=\frac{\mu_{pq}}{{\mu^{\gamma }}_{00}}, \end{array}$$

where

(7) $$\begin{array}{c} \gamma =\frac{p+q}{2}+1\;\text{for}\;\;p+q=2,3,\ldots . \end{array}$$

From the previous equations, the 2D moments invariant to translation, scaling, rotation, and mirroring are derived as follows:

(8) I1=η20+η02,I2=(η20−η02)2+4η112,I3=(η30−3η12)2+(3η21−η03)2,I4=(η30+η12)2+(η21+η03)2,I5=(η30−3η12)(η30+η12)×[(η30+η12)2−3(η21+η03)2]+(3η21+η03)(η21+η03)×[3(η30+η12)2−(η21+η03)2],I6=(η20−η02)[(η30+η12)2−(η102+η03)2]+4η11(η30+η12)(η21+η03),I7=(3η21−η03)(η30+η12)×[(η30+η12)2−3(η21+η03)2]+(3η12−η30)(η21+η03)×[3(η30+η12)2−(η21+η03)2].

From the above, it can be seen that the computational complexity is high for higher-order moments. The invariance of the Hu's image moments for geometrically transformed images can be understood from the illustration in [42].

### 3.3. Triangular Number Generator Function

In the proposed system, we follow a novel approach for generation of Secret Share. Here, we apply a Triangular Number Generator (TNG) function which can uniquely code a pair of integers, to combine the Master Share and the watermark to generate the Secret Share. The mathematical computations to code and recover a pair of integers employing this function appear in [43]. We have applied the same approach in our previous works, to embed a binary logo in the High Frequency (HF) subband and a facial image watermark in the LF subband of CT domain to achieve reversibility and blind extraction. A triangular number is a figurate number which can be represented in a triangular pattern with dots. Triangular numbers are generated by applying (9). This function uniquely encodes a pair of integers (*a*, *b*) into *T* which can be factored back without any overhead

(9) $$\begin{array}{c} T=f\left(a,b\right)=\frac{\left[{\left(a+b\right)}^{2}+3a+b\right]}{2}. \end{array}$$

**Figure 5:**
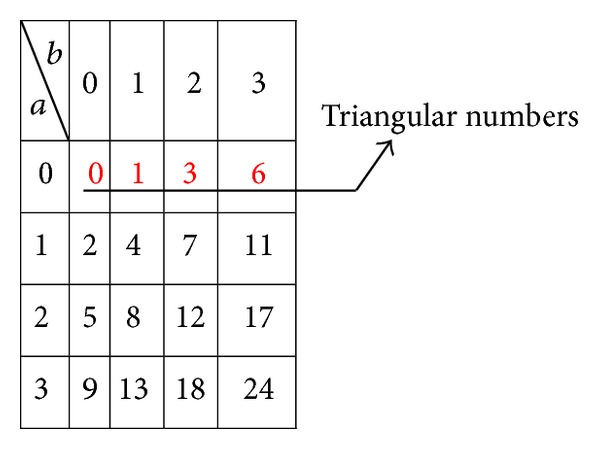
Integer pairs coded with TNG Function.

The *T* values of the coded integer pairs (*a*, *b*) for a small set of values is tabulated in Figure 5. The sequence of triangular numbers appears in the first row of the table. It can be seen that each integer pair is uniquely coded, that is, *f*(*a*, *b*) and *f*(*b*, *a*) are distinct. The integer pair (*a*, *b*) can be restored on applying (10)-(11).

(10) $$\begin{array}{c} C=\frac{\left[\text{sqrt}\left(8T+1\right)-1\right]}{2}, \end{array}$$

where *C* = *a* + *b*

(11) $$\begin{array}{c} a=\frac{T-C\left(C+1\right)}{2},\\ b=\frac{C\left(C+3\right)}{2}-T. \end{array}$$

This approach offers the features of both reversibility and blindness in extraction; that is, *a* and *b* can be recovered exactly without any side information. In the proposed system we have applied (9) for Secret Share generation and (10)-(11) for watermark extraction.

### 3.4. Arnold Transform

Arnold transform is a chaotic transform from the torus onto itself. It can randomize an image and restore it to original form on sufficient number of iterations. Arnold's Map, Duffing Map, Henon Map, and so forth, are common chaotic transforms for the 2D space which are suitable for scrambling and recovering the watermarks. Arnold transform given in (12) is applied to encrypt the embedding position of the host image and the logistic map, to determine the bit positions for embedding in a scheme proposed by Wu and Guan [44]

(12) $$\begin{array}{c} \left[\begin{array}{c} x_{n}\\ y_{n} \end{array}\right]=\left[\begin{array}{cc} 1 & 1\\ 1 & 2 \end{array}\right]\left[\begin{array}{c} x\\ y \end{array}\right]\left(\mathrm{mod}\;n\right). \end{array}$$

In a *n* × *n* spaces any coordinate position (*x*, *y*) can be mapped to (*x*_*n*_, *y*_*n*_) and vice versa on applying the previous equation. Watermark synchronization which refers to locating the position of embedding and extraction is a challenging issue in a watermarking system. The dynamic, invertible, and area-preserving properties of this transform is suitable for realizing synchronization in watermarking systems.

## 4. Proposed System

The EHR, an integrated collection of patient information including demographic information, diagnostic history, clinical findings, laboratory results, and radiology reports. can support the clinicians to provide better medical care. We have suggested the framework and watermarking system for seamless integration of past medical history with radiology readings, focusing on patient authentication and confidentiality. In this section, we present the authentication model and the algorithms for watermark embedding and extraction.

### 4.1. Authentication Framework

**Figure 6:**
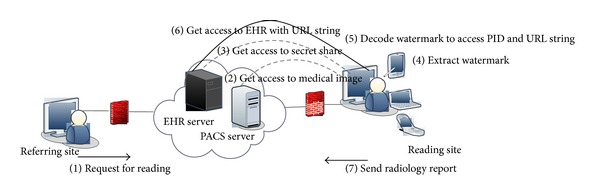
Framework for patient authentication.

The framework is illustrated in Figure 6 and the complete workflow is as follows.

1. Request for reading is sent from the referral site to the remote radiologist.
2. On acceptance, radiologist gets access to the image for study from the Picture Archiving and Communication System (PACS) server.
3. Radiologist gets access to Secret Share from EHR server.
4. Radiologist generates Master Share from the host image and combines with Secret Share to construct watermark.
5. Radiologist decodes the watermark and gets access to PID segment and URL string.
6. Radiologist gets access to EHR of the patient.
7. Radiologist sends the report to the referral site.

### 4.2. Secret Share Creation

The steps for creation of Master Share and Secret Share are given in Algorithm 1.

### 4.3. Watermark Extraction

The steps for watermark construction from the Master Share and Secret Share are given in Algorithm 2. 

**Figure 7:**
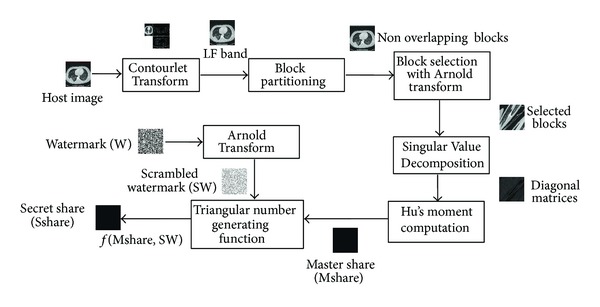
Secret Share creation.

**Figure 8:**
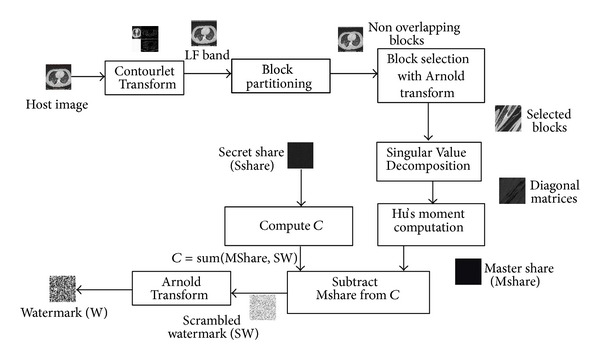
Watermark Construction.

Algorithms 1 and 2 are illustrated in Figures 7 and 8.

Due to its simplicity, the proposed scheme can be deployed in radiology workstations and in hand held devices such as laptops, ipads, and smartphones which provide reliable readings under emergencies.

## 5. Experimental Results 

**Figure 9:**
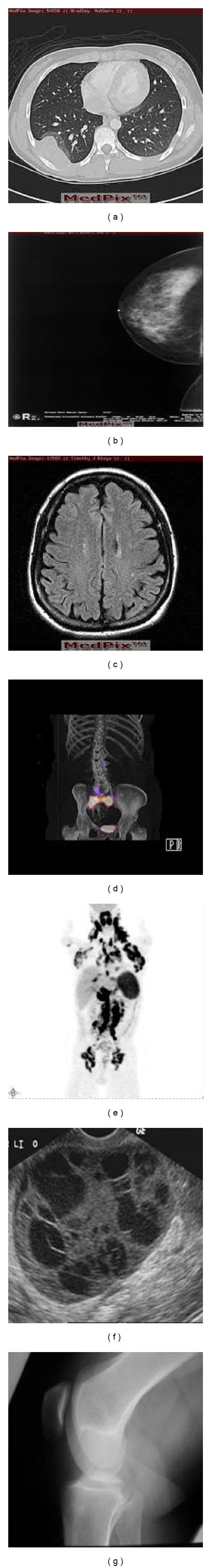
Host Images—(a) CT scan (b) Mammogram (c) MRA (d) Nuclear (e) PET (f) Ultrasound (g) X-ray.

We have implemented the previous algorithms in Matlab 12 software. The algorithms are tested with host images of different modalities such as CT, Mammogram, MRA, PET, Ultrasound, Nuclear, and X-ray each of size 512 × 512 as shown in Figures 9(a)–9(g) and the trimmed watermark of size 77 × 77 in Figure 4(b).

Initially, the host image is subjected to a 1-level CT decomposition to generate an LF band of size 256 × 256. It is divided into 128 × 128 nonoverlapping blocks each of size 2 × 2. The watermark is scrambled on applying the Arnold Transform. For Master Share creation, initially, we have assumed **k**_**i**_ = 32 and **k**_**j**_ = 32; that is, **k** = (32,32) and **i** = 6.

**Figure 10:**
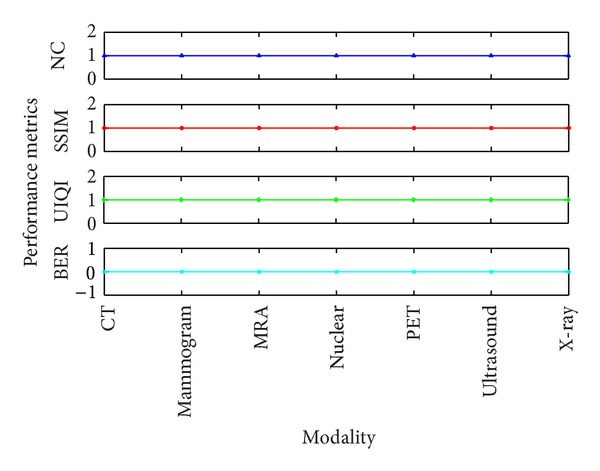
Performance Metrics-Watermark Construction.

With these assumptions, on applying Arnold transform, **k** is mapped to (63,94); that is, for the watermark bit at position (1,1), the Master Share is created out of block (63,94). Subsequently, for each bit in the watermark, **k**_**i**_ and **k**_**j**_ are incremented by 1 to select blocks. The Master Share is combined with the watermark to generate the Secret Share. Similarly, the Master Share is created at the other end following the same procedure. It is combined with the Secret Share to construct the watermark. The extracted watermarks are evaluated with Bit Error Rate (BER), Normalized Correlation coefficient (NC), Structural Similarity Index Measure (SSIM), and Universal Image Quality Index (UIQI) metrics. These performance metrics are shown in Figure 10 for all the modalities. The experimental results show that the watermarks constructed are intact under all modalities.

We have tested the robustness of the watermarks with the checkmark [45] benchmarking software. The extracted watermarks under different attacks are shown in Table 1 with the corresponding BER and NC values. It is evident that the watermark is robust to all classes of attacks.

**Figure 11:**
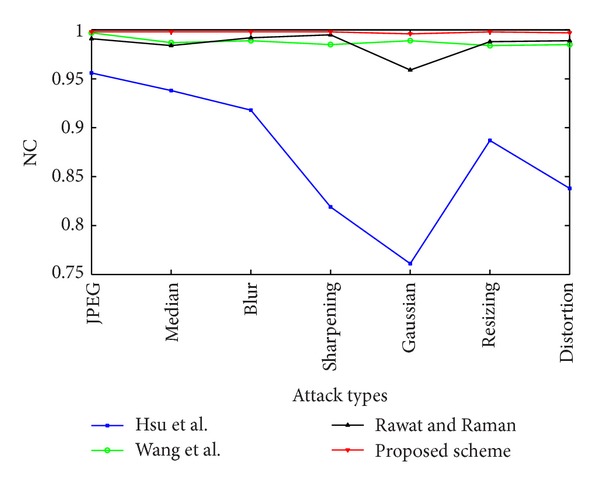
Comparison with existing zero-watermarking schemes.

We have also compared our scheme with those proposed by Hsu and Hou [31], Wang and Chen [32], and Rawat and Raman [33]. For this, we have run the attacks with suitable parameters specified in Rawat and Raman [33] with Matlab software on the host images. The comparison is based on the NC values for a set of attacks under which comparison is made in the later. The results of the attacks are shown in Table 2. The results of comparison are shown in Figure 11. It is evident that the proposed scheme provides better robustness compared to the rest.

We have also compared the proposed scheme with the one proposed by Kim et al. [27] which exclusively embeds a QR code into the spatial, DCT, and FFT domains of the digital image. The comparison is based on the best BER values reported by the authors irrespective of the domain and the embedding strengths. The comparison is shown for JPEG compression, rotation, and shrinking attacks in Tables 3, 4, and 5, respectively. It is seen from the tables that the proposed scheme provides better robustness, invariably for all the modalities. In all the experiments, we have verified that the QR codes are readable.

## 6. Discussion

Robustness to attacks and security are the challenging issues in zero-watermarking systems. In addition to the previous, in the proposed system, the watermarks constructed must also be decodable by a QR code decoder. From the experimental results, it is apparent that the watermarks are robust and readable against a variety of image-processing attacks under different attack parameters. We understand from the embedding and extraction algorithms that the Master Share plays major role in achieving robustness. Here, we have exploited the CT and SVD transform domains and invariant nature of the image moments for Master Share creation. Instead of employing a complete set of image invariants, we have taken only the three lower-order invariants for creating the Master Share. Computational complexity of these invariants is comparatively lower than that of the higher-order invariants. The magnitude of each of these invariants is very small. Here, for ease of computation, we have taken only the sign bits of the invariants. We have considerably reduced the spatial and time complexity by embedding only the kernel of the QR code excluding the quiet zone. The TNG function employed in this scheme offers a provision to resolve false claims of ownership. The Secret Share can be decoded into the Master Share and the watermark blindly without any overhead to prove ownership. The security of the proposed system is attributed to the position of the blocks selected for creation of master and secret shares. In this system, the block selection is based on 2 factors: initial block position and the number of iterations for Arnold Transform to map it to a new position. The area preserving nature of the Arnold Transform presents the freedom of arbitrary block selection. It is highly unlikely that an attacker would able to blindly determine the block positions and generate the Master Share due to the complexity of computations involved. 

From Table 1, we see that the proposed scheme offers robustness even against 75% of cropping. This is ascribed to the stability of magnitude of the moment invariants. We have tabulated the log scaled representation of the Hu's invariants of the unaltered image 9(a) and its cropped versions in Table 6 to understand this. From this table it can be seen that moment magnitudes for the cropped images are closer to that of the original host image in spite of higher degrees of cropping. As we create the Master Shares out of the sign bits of these invariants in the CT-SVD domain, there is no significant variation in them, irrespective of the level of cropping. This in turn attributes to the intactness of the watermark constructed. However, though NC values are similar for the watermarks extracted from the three cropped images, the BER is slightly higher for the one extracted from the image cropped by 75%. 

There are no existing systems proposed for zero-watermarking of QR code particularly for medical images. Though we have tested the system for robustness with a benchmarking software, we have done a fair comparison with similar systems with suitable parameters to establish that our system outperforms the rest. This system for QR based authentication can assist the radiologists to make a better reading; also, it can alleviate medical errors due to mistaken patient identification. Further, the system can be customized to enforce patient consent based EHR sharing in which case; the Secret Share must be possessed by the patient. The proposed system is HITECH compliant as it is designed to provide patient information and access to only authorized radiologists registered with the referral institution. 

## 7. Conclusion

In this paper, we have proposed a framework based on zero-watermarking for patient authentication and controlled access to medical records in a teleradiology environment. The patient identification data encoded in the form of QR code is decodable under all attacks. Comparison with similar techniques shows that the proposed scheme is better in the aspects of resilience, security, and complexity. This system is suitable for implementation in both dayhawk and nighthawk radiology practices for patient authentication, compliant to the requirements of healthcare policies. Further research can be carried out, to tailor the framework to provide fine grained access to different parts of the clinical documents such as EHR, Electronic Medical Records (EMR), Protected Health Information (PHI) records, and Continuity of Care Records (CCR). Extensive studies can be conducted on moment invariants to identify a single unique invariant to be employed in Master Share construction. To supplement the previous, the complexity of the watermarking scheme can be reduced further by embedding only the data and error correction code words of the QR codes.

## Figures and Tables

**Algorithm 1:**
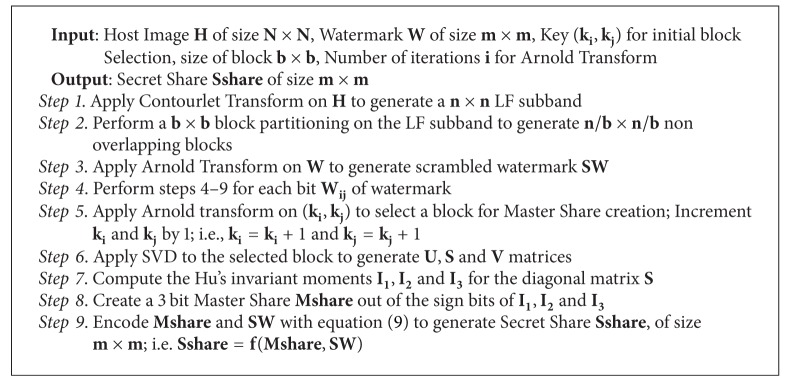
Master share and secret share creation.

**Algorithm 2:**
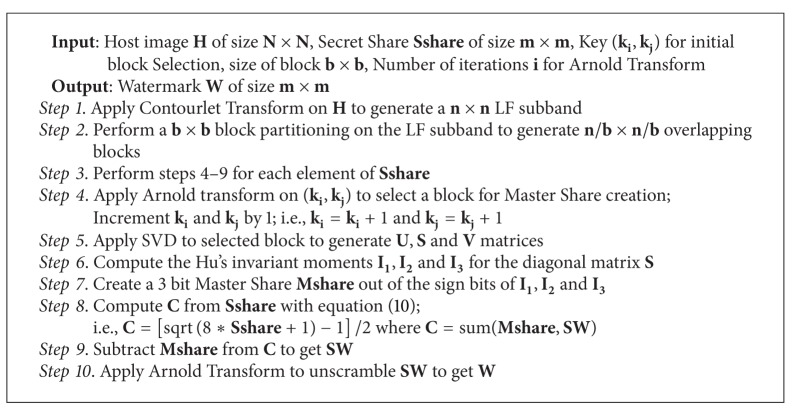
Watermark construction.

**Table 1:** Performance measures for checkmark attacks.

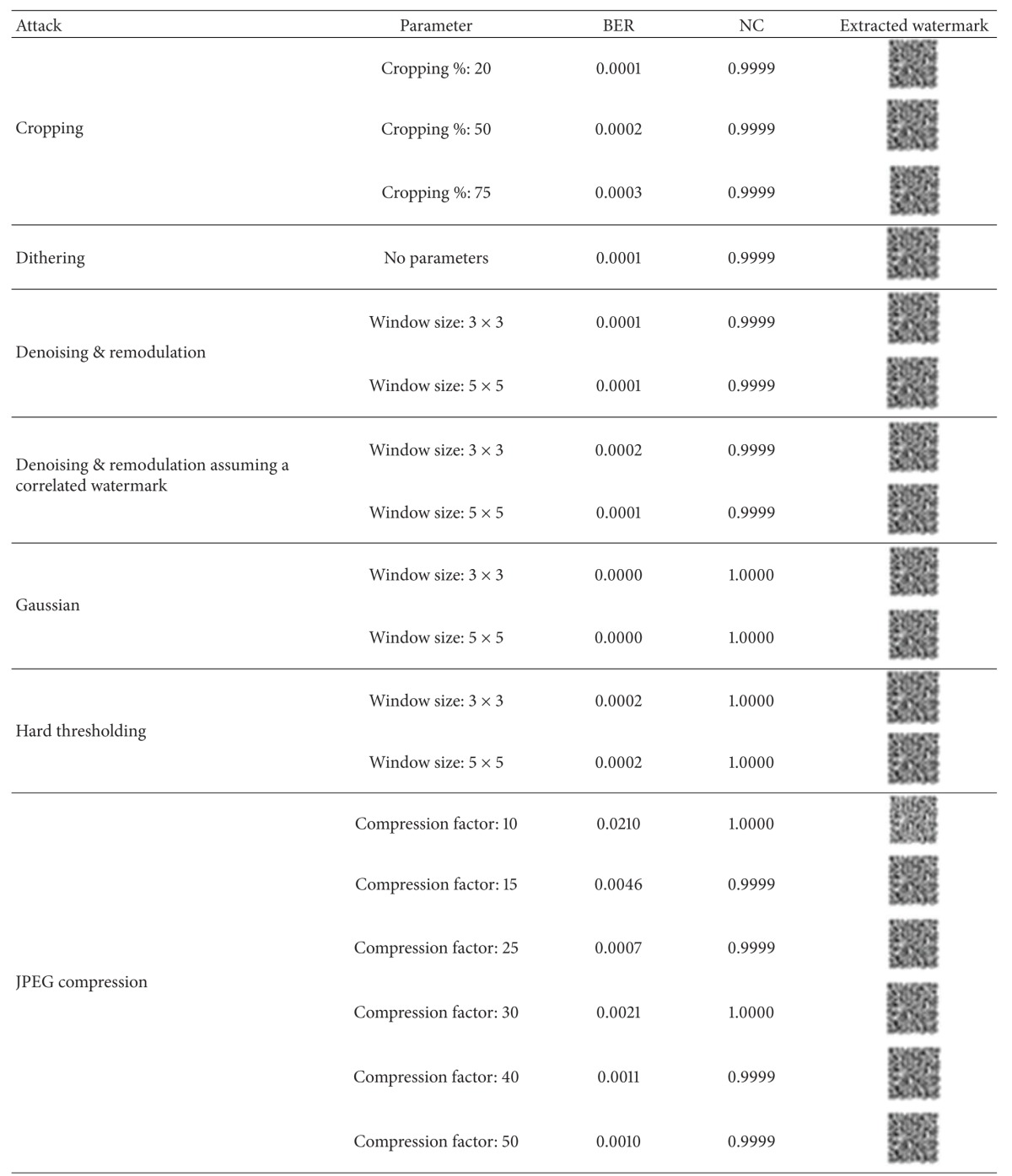 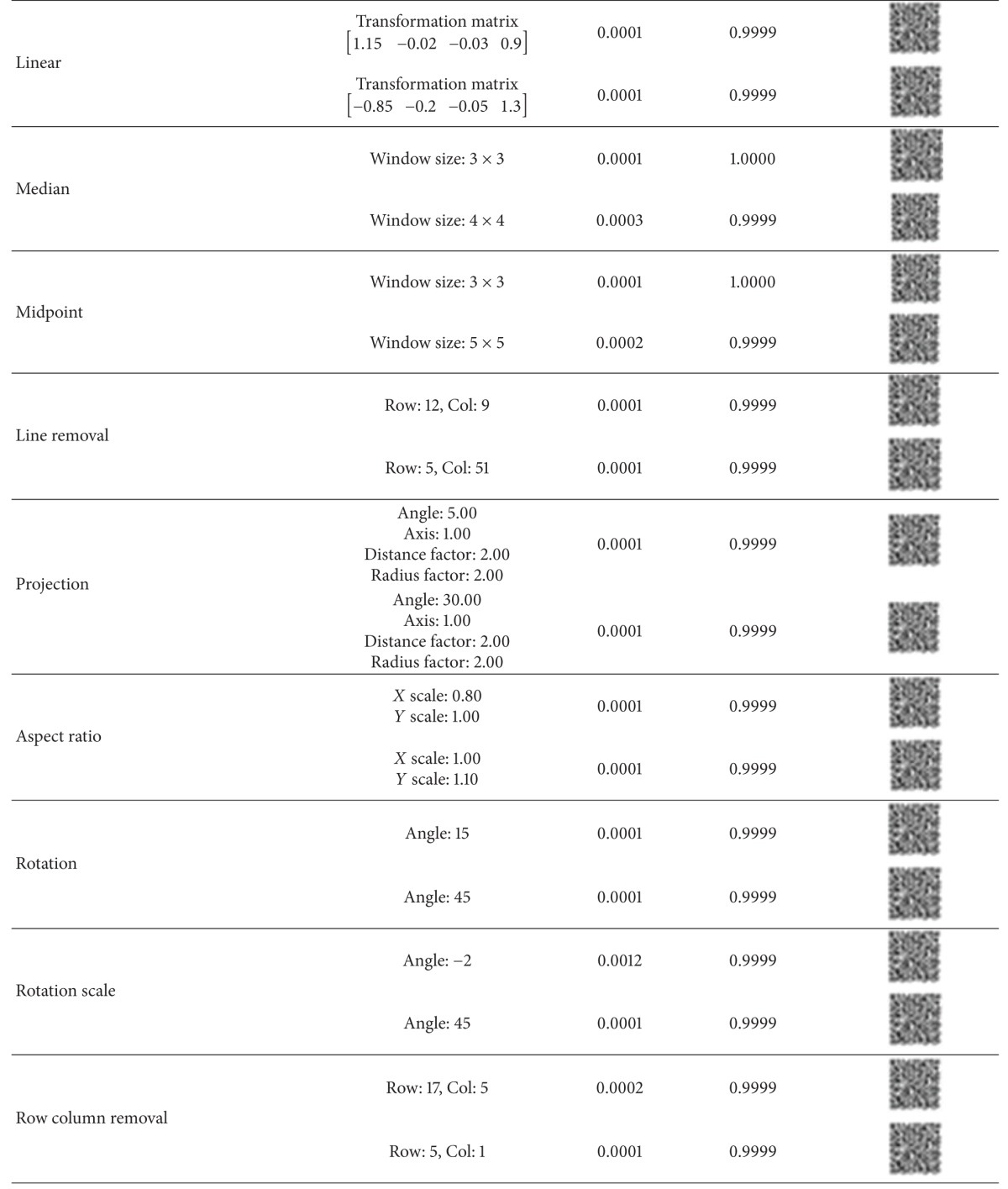 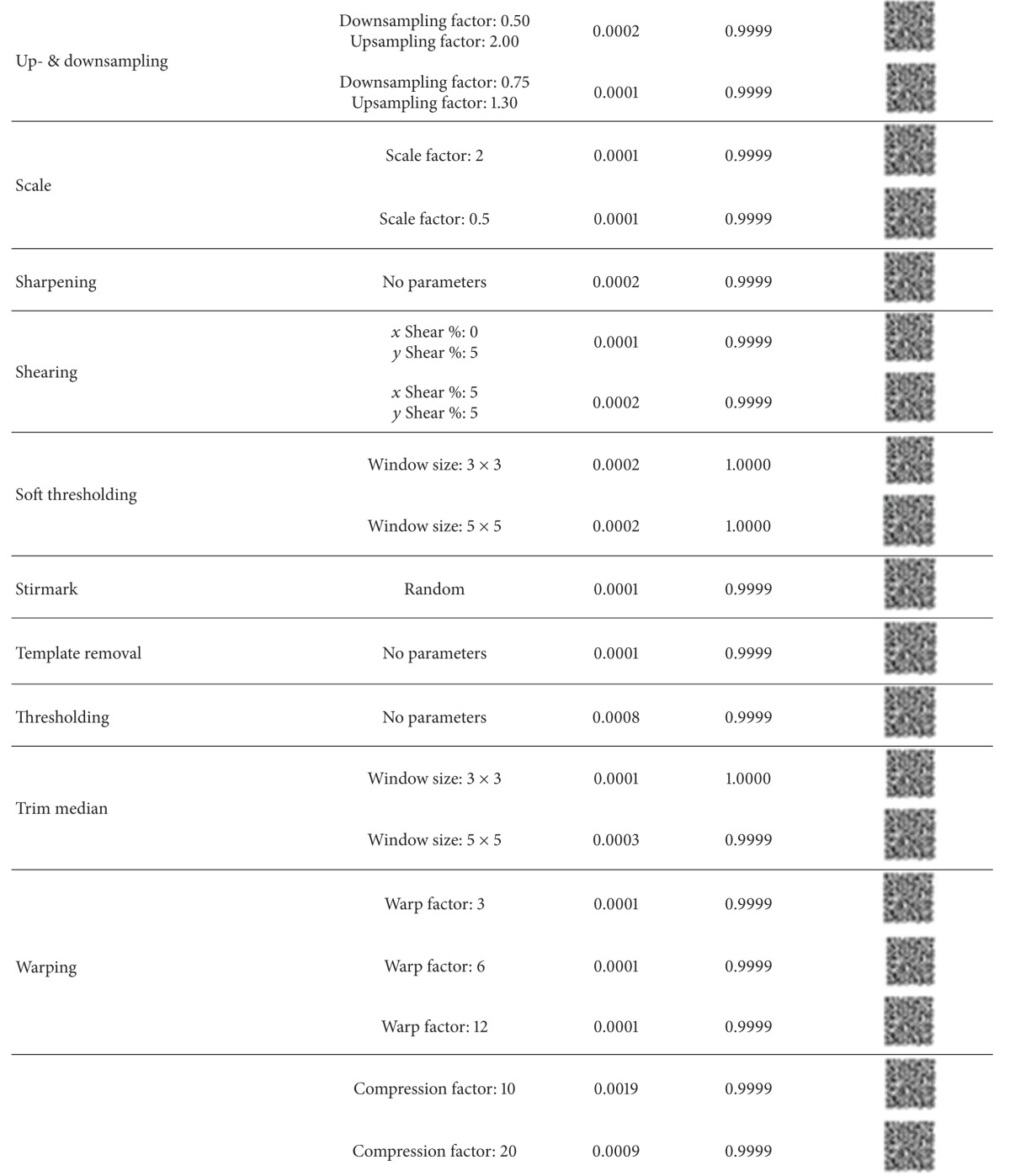 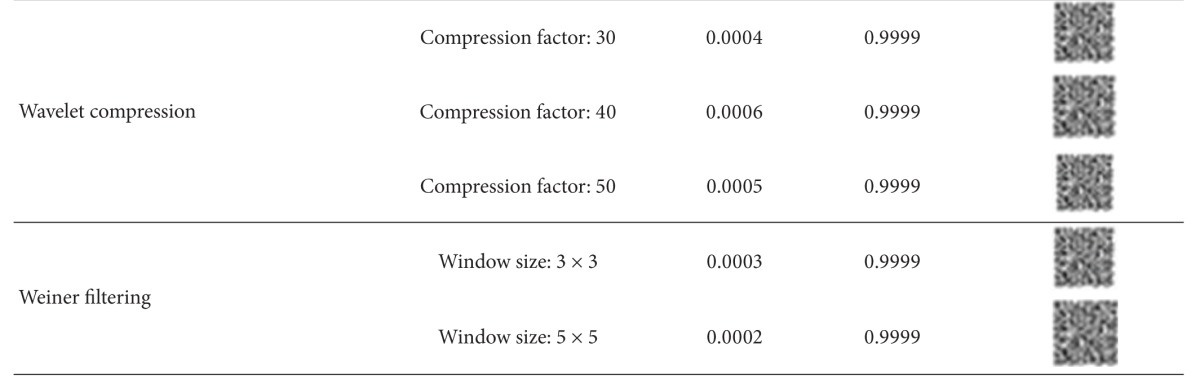

**Table 2:** NC values under Matlab attacks.

Attack	Modality						
	CT	Mammogram	MRA	Nuclear	PET	Ultrasound	X-ray
JPEG compression Quality factor: 50%	0.9999	0.9854	0.9999	1.0000	0.9995	1.0000	0.9990
Average Window size: [9 × 9]	0.9999	0.9891	0.9999	1.0000	0.9995	1.0000	0.9992
Median Window size: [9 × 9]	0.9999	0.9885	0.9997	1.0000	0.9998	1.0000	0.9991
Blur Window size: [9 × 9]	0.9999	0.9885	0.9997	1.0000	0.9997	1.0000	0.9993
Sharpening +50% sharpness	0.9999	0.9875	0.9999	1.0000	0.9997	1.0000	0.9990
Gaussian Noise: 30%	0.9999	0.9755	0.9997	1.0000	0.9994	1.0000	0.9984
Contrast Sharpness: +50%	0.9999	0.9904	0.9998	1.0000	0.9995	1.0000	0.9985
Gamma correction Gamma value: 0.6	0.9999	0.9841	0.9999	1.0000	0.9997	1.0000	0.9993
Histogram Equalization	0.9999	0.9824	0.9998	1.0000	0.9995	1.0000	0.9986
Resizing Scale factor: 0.5	0.9999	0.9804	0.9999	1.0000	0.9994	1.0000	0.9984
Rotation Angle: 3°	0.9999	0.9850	0.9997	1.0000	0.9994	1.0000	0.9985
Distortion Warp factor: 3	0.9999	0.9831	0.9997	1.0000	0.9994	1.0000	0.9988

**Table 3:** Comparison for JPEG compression attacks (BER).

Scheme	Quality factor			
	100%	90%	80%	70%
Kim et al. [27]	0.0732 (Spatial)	0.1953 (Spatial)	4.6143 (Spatial)	12.0850 (Spatial)
Proposed scheme				
CT	0.0001	0.0001	0.0006	0.0005
Mammogram	0.0185	0.0182	0.0253	0.0209
MRA	0.0002	0.0002	0.0002	0.0006
Nuclear	0.0001	0.0001	0.0001	0.0002
PET	0.0004	0.0005	0.0009	0.0010
Ultrasound	0.0001	0.0001	0.0001	0.0001
X-ray	0.0022	0.0018	0.0034	0.0042

**Table 4:** Comparison for rotation attacks (BER).

Scheme	Angle of rotation		
	30°	45°	60°
Kim et al. [27]	17.52 (Spatial)	49.38 (FFT)	4.83 (Spatial)
Proposed scheme			
CT	0.0002	0.0001	0.0001
Mammogram	0.0093	0.0092	0.0064
MRA	0.0002	0.0001	0.0001
Nuclear	0.0006	0.0004	0.0001
PET	0.0005	0.0004	0.0002
Ultrasound	0.0004	0.0001	0.0016
X-ray	0.0011	0.0002	0.0002

**Table 5:** Comparison for shrinkage attacks (BER).

Scheme	Shrinkage %	
	50%	75%
Kim et al. [27]	6.20 (Spatial)	21.58 (Spatial)
Proposed scheme		
CT	0.0009	0.0009
Mammogram	0.0251	0.0239
MRA	0.0006	0.0004
Nuclear	0.0001	0.0002
PET	0.0007	0.0005
Ultrasound	0.0001	0.0001
X-ray	0.0040	0.0034

**Table 6:** Log scaled representation of Hu's moments for original Figure 9(a) and cropped images.